# Immune Monitoring Assay for Extracorporeal Photopheresis Treatment Optimization After Heart Transplantation

**DOI:** 10.3389/fimmu.2021.676175

**Published:** 2021-08-10

**Authors:** Maja-Theresa Dieterlen, Kristin Klaeske, Alexander A. Bernhardt, Michael A. Borger, Sara Klein, Jens Garbade, Sven Lehmann, Francis Ayuketang Ayuk, Herrmann Reichenspurner, Markus J. Barten

**Affiliations:** ^1^Heart Center, HELIOS Clinic, Department of Cardiac Surgery, University Hospital Leipzig, Leipzig, Germany; ^2^Department of Stem Cell Transplantation, University Medical Center Hamburg-Eppendorf, Hamburg, Germany; ^3^Department of Cardiovascular Surgery, University Heart and Vascular Center Hamburg, Hamburg, Germany

**Keywords:** extracorporeal photopheresis, heart transplantation, regulatory T cells, dendritic cells, immune tolerance

## Abstract

**Background:**

Extracorporeal photopheresis (ECP) induces immunological changes that lead to a reduced risk of transplant rejection. The aim of the present study was to determine optimum conditions for ECP treatment by analyzing a variety of tolerance-inducing immune cells to optimize the treatment.

**Methods:**

Ten ECP treatments were applied to each of 17 heart-transplant patients from month 3 to month 9 post-HTx. Blood samples were taken at baseline, three times during treatment, and four months after the last ECP treatment. The abundance of subsets of tolerance-inducing regulatory T cells (T_regs_) and dendritic cells (DCs) in the samples was determined by flow cytometry. A multivariate statistical model describing the immunological status of rejection-free heart transplanted patients was used to visualize the patient-specific immunological improvement induced by ECP.

**Results:**

All BDCA^+^ DC subsets (BDCA1^+^ DCs: p < 0.01, BDCA2^+^ DCs: p < 0.01, BDCA3^+^ DCs: p < 0.01, BDCA4^+^ DCs: p < 0.01) as well as total T_regs_ *(*p < 0.01) and CD39^+^ T_regs_ *(*p < 0.01) increased during ECP treatment, while CD62L^+^ T_regs_ decreased (p < 0.01). The cell surface expression level of BDCA1 (p < 0.01) and BDCA4 (p < 0.01) on DCs as well as of CD120b (p < 0.01) on T_regs_ increased during the study period, while CD62L expression on T_regs_ decreased significantly (p = 0.04). The cell surface expression level of BDCA2 (p = 0.47) and BDCA3 (p = 0.22) on DCs as well as of CD39 (p = 0.14) and CD147 (p = 0.08) on T_regs_ remained constant during the study period. A cluster analysis showed that ECP treatment led to a sustained immunological improvement.

**Conclusions:**

We developed an immune monitoring assay for ECP treatment after heart transplantation by analyzing changes in tolerance-inducing immune cells. This assay allowed differentiation of patients who did and did not show immunological improvement. Based on these results, we propose classification criteria that may allow optimization of the duration of ECP treatment.

## Introduction

Since the first report in 1991, the American Society of Apharesis recommends extracorporeal photopheresis (ECP) for the treatment of acute cellular and recurrent rejection (ACR) as well as for rejection prophylaxis after heart transplantation (HTx) (1). Additionally, experts in the field of transplantation medicine recommend chronic ECP treatment of HTx patients with donor specific antibodies (DSA) (2). Although ECP has been used to treat an increasing number of patients in recent years, there is still no consensus about the optimal ECP therapy for any individual patient. For example, questions remain about the best time point to initiate or reintroduce ECP therapy as well as about the optimal number of ECP treatments that are required for different indications, such as ACR or antibody-mediated rejection (AMR). Thus, a reliable monitoring tool for optimizing ECP therapy is required (3).

Based on the results of our previous ECP studies, we proposed that monitoring specific immune cells during ECP treatment might provide information that could be used to optimize ECP treatments (3, 4), which has been mentioned in the updated European Dermatology Forum on the use of extracorporeal photopheresis (5). Currently, two different mechanisms of action are discussed for ECP therapy. One hypothesizes that the return of apoptotic T cells activates dendritic cells (DCs), which leads to cytokine alterations and results in an increase in regulatory T cells (T_regs_) (6). The other hypothesizes that ECP presents an apoptotic stimulus that affects activated alloreactive T cells, which are preferentially processed and presented by DCs resulting in suppression of alloantigen-responding T cells (3). In particular, the effect of ECP on an increase of T_regs_ in HTx was studied by different research groups (7–11).

In previous studies, we showed that T_reg_ and DC subsets in HTx patients with different indications for ECP treatment, such as prophylactic treatment, ACR, or cardiac allograft rejection (CAV) responded differently to ECP (3, 4).

Thus, for the current study we proposed that analysis of the expression of DCs and T_regs_ in peripheral blood could be helpful in designing a monitoring tool for ECP. We validated our immune cell assays to differentiate between patients with and without immunological effects after ECP therapy. Ideally, such a monitoring tool should allow optimization of individual ECP treatment schedules and reduce or prolong ECP treatments depending on the immunological effects.

## Materials and Methods

### Patient Cohorts

The study cohort included 17 patients aged over 18 years who received HTx between May 2016 and January 2018 at the Department of Cardiovascular Surgery of the University Heart and Vascular Center in Hamburg, Germany. Four patients were excluded because they refused to receive ECP. In accordance with the recommendations of the ECP guidelines of both, the American Apharesis Society and the European Dermatology Forum, the patients were classified into two study groups (5, 12). The first group consists of patients who had no rejection before ECP start (prophylactic treatment) and the second group had an AMR or ACR before ECP start (rejection treatment). Therefore, patients of both study groups received ECP as chronic treatment to avoid rejection. Written informed consent was obtained from each participant before initiation of ECP (vote no. PV7246, Ärztekammer Hamburg, Germany) in accordance with the Declaration of Helsinki and the local ethical regulations. Patient demographics, disease and treatment parameters as well as their immunosuppressive regimens were documented.

### Extracorporeal Photopheresis

**Figure 1:**
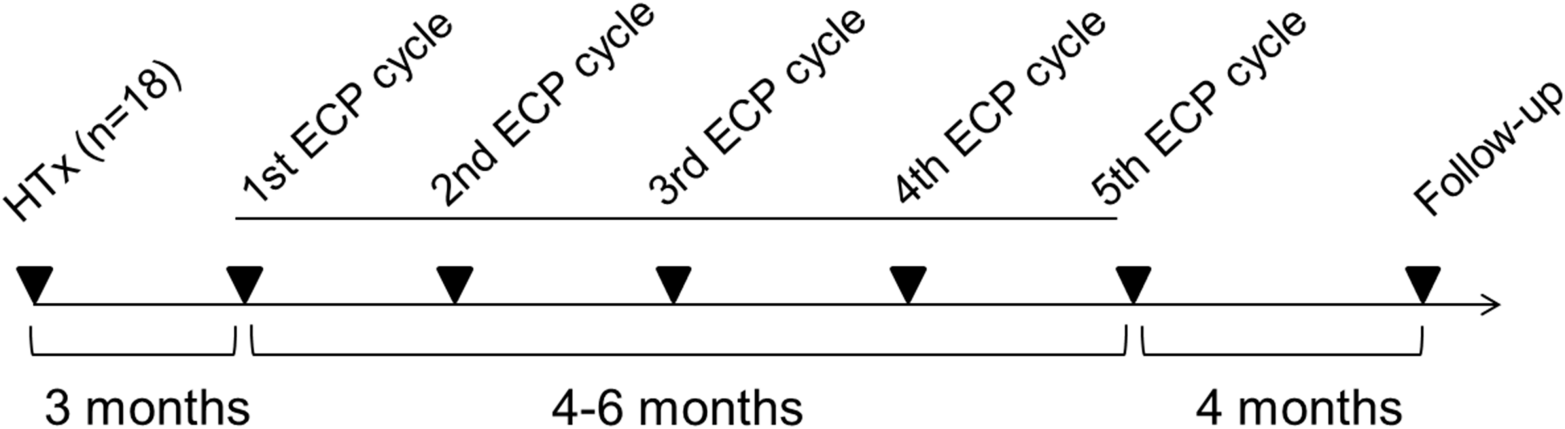
Overview about the treatment regimen for extracorporeal photopheresis. ECP, extracorporeal photopheresis; HTx, heart transplantation.

ECP was performed using the closed inline THERAKOS CELLEX photopheresis system (Therakos Inc., West Chester, PA, USA) with a total of ten ECP treatments that were grouped to five ECP cycles. ECP treatments were conducted on two consecutive days, and ECP cycles were performed every 4-6 weeks (**Figure 1**). The daily ECP procedure included separation of peripheral blood mononuclear cells (PBMCs) by centrifugation of the patient’s whole blood. Following centrifugation, the remaining blood components were reinfused immediately. PBMCs were sequentially exposed to 8-methoxypsoralen (20 µg/mL) and ultraviolet A light (~1.5 J/cm^2^). Photoactivation time and the entire cell volume were automatically calculated using the patient’s hematocrit in the buffy coat by integrated software in the Cellex^©^ ECP machine. After completion of the photoactivation process, PBMCs were immediately re-transfused to the patient. Blood count analysis after reinfusion of the ECP product was performed by documenting platelet and erythrocyte count, and the hemoglobin and hematocrit content.

### Flow Cytometric Assessment

Phlebotomy was performed before each ECP cycle and a follow-up blood analysis was conducted four months after the last ECP cycle. Peripheral blood samples obtained from the patients were treated as described previously (3). T_regs_ were defined as CD3^+^/CD4^+^/CD25^high^/CD127^low^ cells; from this population, the T_reg_ subsets expressing CD39, CD62L, CD120b or CD147 were analyzed. Subsets of DCs were quantified by stainings using lineage cocktail-1, HLA-DR, and blood dendritic cell antigen (BDCA) 1, 2, 3 or 4. Antibodies were obtained from Becton Dickinson (BD, Heidelberg, Germany) or BioLegend (Fell, Germany). For each staining, 200 µl (T_reg_ analysis) or 300 µl (DC analysis) of human heparinized whole blood were mixed with the appropriate antibody cocktail and incubated for 20 min at room temperature in the dark. Next, 2 ml of FACS lysing solution (BD) were added and samples were incubated for 10 min. After centrifugation at 300x g for 5 min, the supernatant was discarded and samples were washed with 4 ml phosphate-buffered saline (PBS). The supernatant was discarded after washing, and the cells were fixed with 500 µl 1% formaldehyde-PBS solution. Samples were analyzed directly using a *BD LSR II Flow Cytometer* and *BD FACSDiva version 6.1.3* software (both BD); 10,000 events of CD3^+^CD4^+^ cells (for T_reg_ analysis) and 500,000 vital cells (for DC analysis) were analyzed per sample. Mean fluorescence intensities (MFIs) were documented for BDCA1-4 on DCs as well as for CD39, CD62L, CD120b, and CD147 on T_reg_ subsets.

### Statistics

The patient cohort was characterized by mean (± standard deviation) for continuous and by number (percent) for categorical variables. Time-dependent changes of cellular parameters were analyzed by the generalized linear model for repeated measurements. A simple contrast was used, and the first measurement (pre ECP) was set as the reference. Tests were performed two-sided at 5% significance level. All analyses were done using Intel SPSS Statistics version 23 (IBM Corp. 1989, 2011).

We combined immune markers that are involved in tolerance induction after ECP to describe the patient’s immune transplant tolerance phenotype. The immune phenotype is defined as the percentage of tolerance-inducing immune cells and is called immunological profile. A valid statistical tool to perform this systemic analysis of immune profiles is the hierarchical clustering which has been performed in previous clinical studies for comparable analyses (12, 13). Hierarchical cluster analysis using the ClustVis software (Bioinformatics, Algorithms and Data Mining Group, University of Tartu, Estonia) was performed for every ECP-treated patient in combination with the dataset described in the recent work of Klaeske et al. (13) Five flow cytometric parameters of DCs (% total DCs/PBMCs, % BDCA1^+^ DCs/total DCs, % BDCA2^+^ DCs/total DCs, % BDCA3^+^ DCs/total DCs and % BDCA4^+^ DCs/total DCs) and six parameters of T_regs_ (% CD4^+^ T cells/total T cells, % T_regs_/CD4^+^ T cells, % CD39^+^ T_regs_/total T_regs_, % CD62L^+^ T_regs_/total T_regs_, % CD120b^+^ T_regs_/total T_regs_ and % CD147^+^ T_regs_/total T_regs_) were included in the cluster analysis. The hierarchical cluster analysis leads to the pattern recognition of a tolerance-inducing phenotype and displays the distance connectivity of the immunological profile for every measurement of an ECP-treated patient. As a result, it is possible to monitor whether an ECP-treated HTx patient develops a tolerance-promoting immunological phenotype. This tool could be helpful for clinicians to monitor, to shorten or prolong the ECP schedule for patients depending on the immunological profile. Patient-specific results could be available 4-5 hours following blood withdrawal.

### Classification of Immunological Effects Induced by ECP

A classification system for the objective evaluation of immunological effects induced by ECP was established. The hierarchical cluster analysis of the dataset reported by Klaeske et al. (14) formed two clusters. The first cluster included 75% long-term HTx patients and the second cluster included 67% pre-HTx patients (**Supplementary Figure 1**). It can be assumed that stable long-term transplanted patients who never suffered from transplant rejection received an optimal immunosuppression and have an immune phenotype promoting transplant tolerance. Klaeske et al. used hierarchical clustering and principle component analyses to show that this immune phenotype of long-term HTx patients differed from that of pre-HTx patients (14). A hierarchical cluster analysis including the dataset of the previous study from Klaeske et al. and measurements of an ECP-treated patient will allow to evaluate if the immune phenotype of the ECP-treated patients changes to the transplant tolerance immune phenotype during ECP treatment by changes of the position in the heat map of the cluster analysis towards the cluster consisting of long-term HTx patients. The patient-specific reference point in the heat map was the measurement prior to ECP treatment. Every subsequent immunological measurement during and after ECP produced a new point in the heat map. An immunological improvement existed if the measurement shifted toward the cluster containing the majority of long-term HTx-patients. Thus, patients were classified into five categories according to the time point of immunological improvement during ECP (category A: improvement after the 1^st^ ECP cycle, category B: improvement after the 3^rd^ ECP cycle, category C: improvement after the 5^th^ ECP cycle, category D: improvement after the 5^th^ ECP cycle, but declining in the follow-up period, category E: no improvement).

## Results

### ECP Performance and Blood Monitoring

The study cohort consisted of n = 17 HTX patients (11 male, 6 female) with a mean age of 48.8 ± 10.8 years and a mean body mass index of 25.7 ± 5.8 kg/m^2^). The etiology for HTx was dilated cardiomyopathy (n = 11), ischemic cardiomyopathy (n = 4) or had other reasons (n = 2). All patients received a triple-drug immunosuppressive regimen at study begin, whereas n = 10 patients received tacrolimus/everolimus/steroids, n = 4 patients received tacrolimus/mycophenolic acid/steroids and n = 3 patients received everolimus/mycophenolic acid/steroids. The indication for ECP treatment was an existing ACR or AMR (n = 6), and a prophylactic treatment (n = 11). ECP treatment was performed according to the manufacturer’s instruction and was accompanied by blood cell counts of erythrocytes and platelets as well as the hematocrit and hemoglobin content as quality control metrics. Platelet count (p = 0.24), erythrocyte count (p = 0.57), hemoglobin content (p = 0.92), and hematocrit (p = 0.81) did not change significantly during ECP or the follow-up period (**Table 1**). However, the hemoglobin content of the ECP-treated patients was below the hemoglobin reference value (men: 13.5 ± 17.5 g/dL, women: 12.0 ± 15.5 g/dL). In two patients with prophylactic ECP treatment, ACR episodes occurred during ECP treatment. One patient (female, 34 years old) had a higher immunological risk due to two pregnancies and chronic left ventricular assist device therapy before HTx. She got an ACR of a histological grade 3R (ISHLT 2004). The other patient (male, 63 years old) suffered from an early cytomegalovirus infection in the first month post-HTx and got an ACR of histological grade 2R (ISHLT 2004). The ACRs were without hemodynamic compromise, and, therefore, both patients were treated with methylprednisolone (total of 3000 mg) in addition to ECP as well as with an increase of both the tacrolimus and everolimus exposure at time of diagnosis of rejection (month 2 and month 3 after ECP start, respectively). Both patients completed the scheduled ECP treatments. At the end of the study the female patient had a grade 1R ACR and the male patient had no ACR.

**Table 1:** Blood parameters of patients treated with extracorporeal photopheresis.

		Extracorporeal photophoresis				p
	Pre-ECP	1^st^ cycle	3^rd^ cycle	5^th^ cycle	ECP FU	
**Platelets [10^9^/L]**	191 ± 56	238 ± 88	226 ± 67	222 ± 91	217 ± 93	0.24
**Erythrocytes [10^9^/L]**	3.9 ± 0.7	3.8 ± 0.6	4.3 ± 0.6	4.3 ± 0.5	4.3 ± 0.7	0.57
**Hemoglobin [g/dL]**	10.4 ± 1.5	10.2 ± 1.2	11.6 ± 1.4	11.6 ± 1.3	11.6 ± 1.9	0.92
**Hematocrit [%]**	31.9 ± 4.6	31.7 ± 3.3	35.4 ± 4.1	35.9 ± 3.5	34.8 ± 5.4	0.81

pre-ECP, prior extracorporeal photopheresis; ECP FU, follow-up of extracorporeal photopheresis.

### Dendritic Cell Analysis

**Figure 2:**
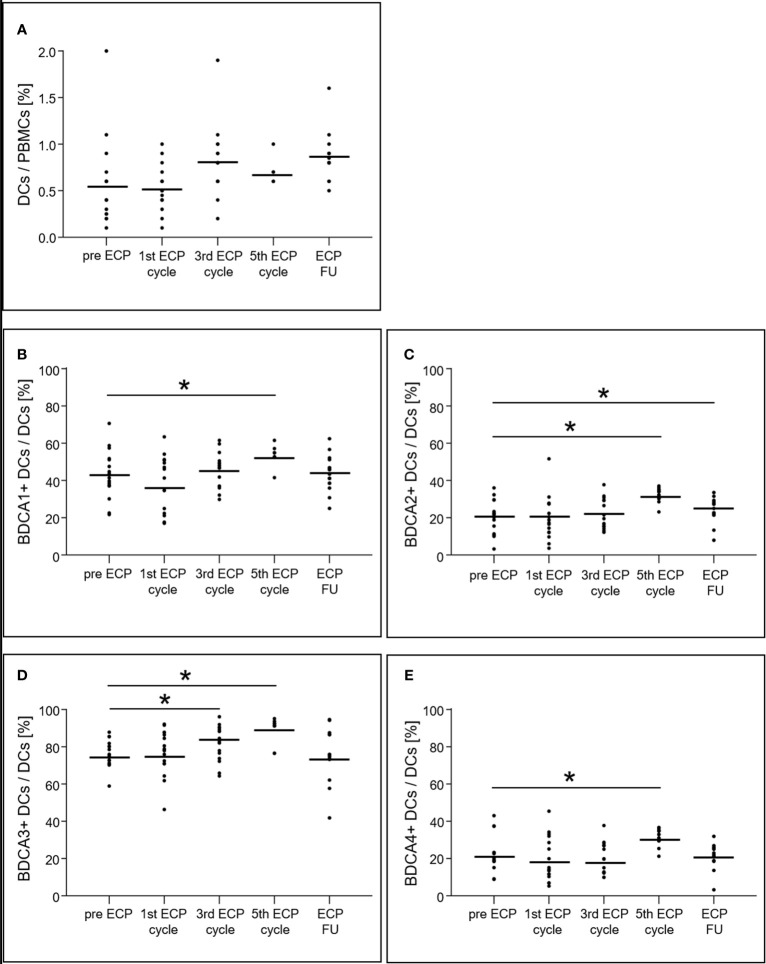
Expression of dendritic cells **(A)** and their subsets **(B–E)** in heart-transplanted patients receiving extracorporeal photopheresis. * marks significant differences (p ≤ 0.05); BDCA1/2/3/4, blood dendritic cell antigen 1/2/3/4; DCs, dendritic cells; ECP, extracorporeal photopheresis; FU, follow-up; PBMCs, peripheral blood mononuclear cells.

While the percentage of total DCs on PBMCs did not change significantly during ECP (p = 0.24; **Figure 2A**), differential consideration showed that all BDCA^+^ DC subsets increased during ECP treatment (BDCA1^+^ DCs: p < 0.01, BDCA2^+^ DCs: p < 0.01, BDCA3^+^ DCs: p < 0.01, BDCA4^+^ DCs: p < 0.01), but decreased to values observed prior to ECP in the follow-up period (BDCA1^+^: pre-ECP 43.0 ± 12.6%, ECP follow-up 43.7 ± 9.1%; BDCA2^+^: pre-ECP 20.5 ± 8.6%, ECP follow-up 23.9 ± 6.3%; BDCA3^+^: pre-ECP 76.2 ± 7.1%, ECP follow-up 75.6 ± 13.1%; BDCA4^+^: pre-ECP 21.9 ± 9.3%, ECP follow-up 21.3 ± 6.3%) (**Figures 2B–E**).

**Figure 3:**
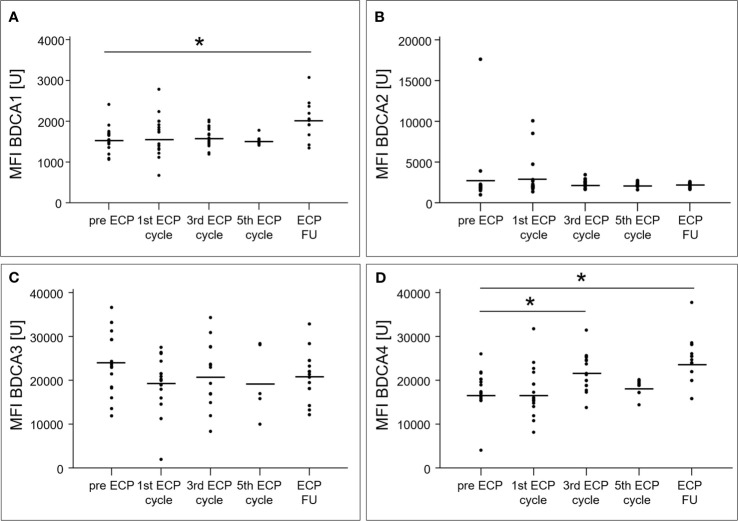
Mean fluorescence intensities of blood dendritic cell antigens 1 **(A)**, 2 **(B)**, 3 **(C)** and 4 **(D)** of dendritic cells in heart-transplanted patients receiving extracorporeal photopheresis. * marks significant differences (p ≤ 0.05); BDCA1/2/3/4, blood dendritic cell antigen 1/2/3/4; ECP, extracorporeal photopheresis; FU, follow-up; MFI, mean fluorescence intensity; U, unit.

An increase of the surface expression level estimated by mean fluorescence intensity, was detected in the follow-up period for BDCA1 (2028 ± 389 U, p < 0.01) as well as after the third ECP cycle (22079 ± 4265 U, p < 0.01) and in the follow-up period (24212 ± 5172 U, p < 0.01) for BDCA4 (**Figures 3A, D**). The surface expression levels of BDCA2 (p = 0.47) and BDCA3 (p = 0.22) were unaffected (**Figures 3B, C**).

An overview about the DC analysis for ECP-treated patients with ACR or AMR as well as for patients treated prophylactically with ECP was presented in **Supplementary Table 1**.

### Regulatory T Cell Analysis

**Figure 4:**
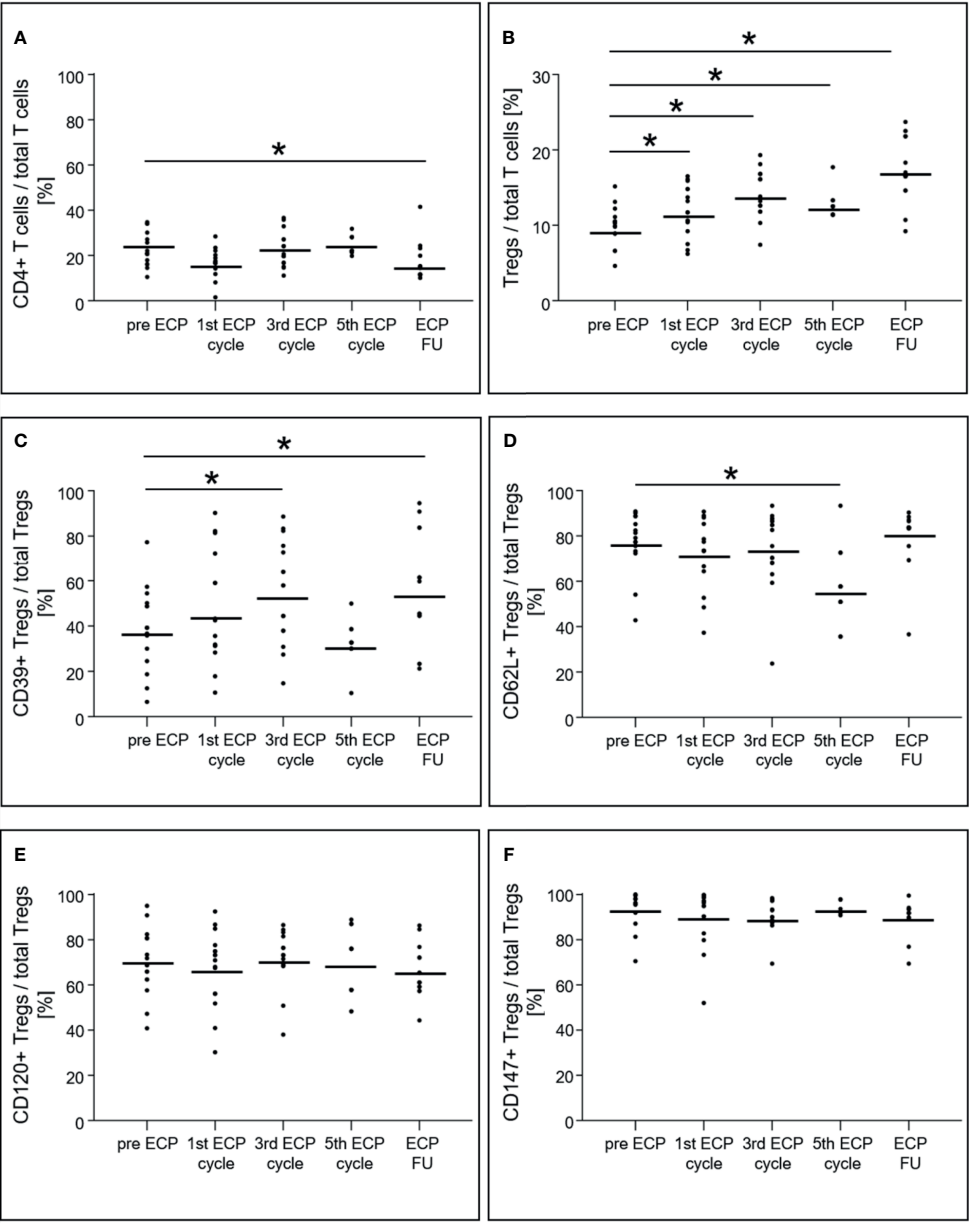
Expression of CD4^+^ T cells **(A)**, regulatory T cells **(B)** and their subsets **(C–F)** in heart-transplanted patients receiving extracorporeal photopheresis. * marks significant differences (p ≤ 0.05); CD, cluster of differentiation; ECP, extracorporeal photopheresis; FU, follow-up; T_regs_, regulatory T cells.

**Figure 5:**
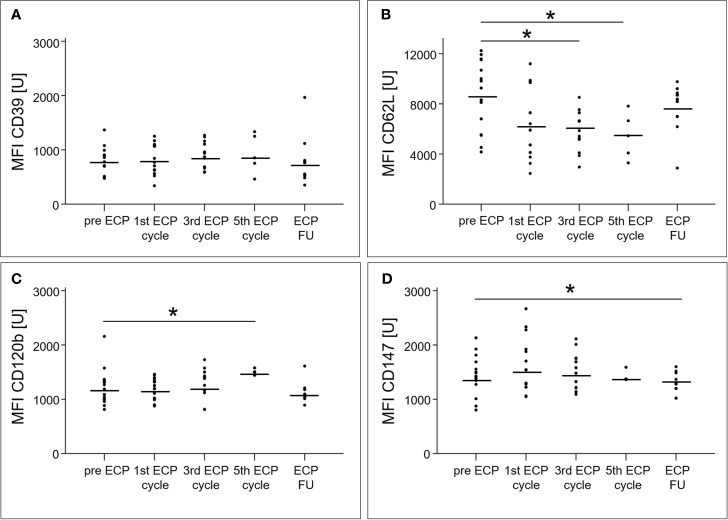
Mean fluorescence intensities of the surface molecules CD39 **(A)**, CD62L **(B)**, CD120b **(C)** and CD147 **(D)** of regulatory T cell subsets in heart-transplanted patients receiving extracorporeal photopheresis. * marks significant differences (p ≤ 0.05); CD, cluster of differentiation; ECP, extracorporeal photopheresis; FU, follow-up; MFI, mean fluorescence intensity; U, unit.

The percentage of CD4^+^ T cells among total T cells decreased from 22.8 ± 7.2% prior to ECP to 16.2 ± 7.8% in the follow-up period (p < 0.01, **Figure 4A**), while the percentage of T_regs_ in the CD4^+^ T cell population increased from 9.9 ± 2.5% to 17.7 ± 4.2% (p < 0.01, **Figure 4B**). The T_reg_ subset expressing CD39 increased within the T_reg_ population during ECP (pre-ECP: 38.5 ± 17.4%, third ECP cycle: 54.6 ± 21.6%) and throughout the follow-up period (54.9 ± 22.9%, p < 0.01; **Figure 4C**). The CD62L^+^ T_regs_ decreased during ECP from 77.2 ± 12.5% prior to ECP to 56.0 ± 14.4% after the fifth ECP cycle (p < 0.01), while CD120b^+^ (p = 0.56) and CD147^+^ T_regs_ (p = 0.48) remained constant (**Figures 4D–F**). The expression of CD39 (p = 0.14) and CD147 (p = 0.08) on the surface of T_regs_ was unchanged during ECP treatment (**Figure 5**). While the surface expression of CD62L decreased during ECP (pre-ECP: 8606 ± 2617 U, third ECP cycle: 5979 ± 1452 U, fifth ECP cycle: 5459 ± 1843 U, p = 0.04), CD120b expression increased significantly at the end of the ECP treatment (pre-ECP: 1199 ± 319 U, fifth ECP cycle: 1496 ± 31 U, p < 0.01).

An overview about the T_reg_ cell analysis for ECP-treated patients with ACR or AMR as well as for patients treated prophylactically with ECP was presented in **Supplementary Table 2**.

### Grouping According to Immunological Profiles

To monitor the patient-specific success of ECP treatment, monitoring data from each ECP-treated patient were combined with a dataset generated by a previous study comprising pre-HTx and long-term HTx patients and were evaluated by cluster analysis. The individual immunological improvement of the ECP treatment was classified according to the time-point of immunological upgrade towards the long-term HTx configuration of tolerance-inducing cell subsets. Exemplary classifications are shown in **Supplementary Figures 1**–**3**.

The patient-specific cluster analyses identified immunological improvement for six patients in the category A, four patients in the category B, and three patients in the category C. For these 13 HTx patients (72%), ECP treatment led to an immunological improvement during ECP and throughout the follow-up period. Ten of the 17 ECP-treated patients (56%) were classified into category A or B and showed an immunological improvement in the latest after three ECP cycles. For the patients in category D (n = 4) and E (n = 1), the immunological efficacy of ECP treatment is questionable. However, clinical outcome measurements were not included in the current study.

## Discussion

The Guidelines on the Use of Therapeutic Apheresis in Clinical Practice stated that ECP treatments after HTx should be continued until stabilization of symptoms or improvement of cardiac function, biopsy findings or donor-specific antibody levels (15). Although these goals are of paramount interest, the duration of ECP therapy required differs from individual to individual and from indication to indication. Thus, the purpose of our study was to develop a classification system based on the immunological effects of ECP to support clinical decisions regarding the optimal number of ECP treatments for HTx patients. However, a proof-of-concept was not part of the present study.

Overall, the clinical efficacy of ECP therapy in this study was high and in line with published data from the landmark trial of Barr et al. who showed a significant reduction of rejection episodes in patients treated with ECP as compared to the control patients (16). Our results show that monitoring DC and T_regs_ expression in peripheral blood might qualify to analyze patient-specific ECP effects. Furthermore, we combined this immune cell monitoring with a multivariate analysis of ECP-induced effects. The basis of this analysis was a multiparametric setting of immune cell subsets involved in tolerance induction. To present the multidimensionality of the immune system, we analyzed eleven parameters and, amended the statistical model accordingly; we also performed cluster analysis with a hierarchical clustering algorithm; data preprocessing and modification was avoided. This statistical method is useful for unusual similarity measures and extracts useful information from larger datasets with many groups (17).

The ECP-induced increase of T_regs_ (4, 7–10, 18) and pDCs (4, 19) has been demonstrated in several studies of heart and lung transplant patients as well as of patients suffering from graft-versus-host disease. Previous work from our group showed that it is possible to differentiate between ECP-treated patients with a “positive ECP immunological effect” and “no ECP effect” (4). The present study refined those observations, including a more detailed cell subset analysis and a more eligible statistical methodology to handle a multivariate dataset.

All BDCA^+^ subsets of DCs increased during ECP, but only the percentage of BDCA2^+^ DCs remained high after ECP. Furthermore, an increased surface expression for BDCA1^+^ and BDCA4^+^ DCs was induced by ECP and was detected by analysis of the MFIs. BDCA2 is a pDC-specific transmembrane lectin that inhibits induction of interferon-α/β, thereby preventing a Th1-type immune response (20). Thus, it can be hypothesized that one mechanism of action of ECP treatment is the suppression of Th1-type immune responses *via* inhibition of interferon-α/β by pDCs. This example clearly demonstrates that immunological monitoring can help to further clarify the mechanism of action of ECP and could uncover unknown cellular effects.

In contrast to difficulties in interpreting increased surface expression of BDCA1 in the context of tolerance induction, the increase of BDCA4 expression in DCs is an observation of great interest. It has been reported that BDCA4, also known as neuropilin-1, can be transferred from DCs to T cells *via* trogocytosis (21), and, therefore, could be detected in natural T_regs_ with a proven suppressive function (22). Thus, the increase of BDCA4 expression during and after ECP treatment detected in our study could be associated with the induction of tolerance in ECP treated patients. This hypothesis is reinforced by the findings of a murine transplantation study that indicated a suppressive role of CD4^+^/BDCA4^+^ T cells (23). Furthermore, a reduction of BDCA4^+^ cells in kidney transplant biopsies was observed during acute rejection compared to those in non-rejecting individuals (24).

Besides the ECP-induced changes in DC subsets, the composition of T_reg_ subsets exhibited substantial modifications during and after ECP treatment. The measured effects documented in earlier reports of ECP treatment were limited to the increase of the total T_reg_ population and the highly suppressive CD39^+^ T_reg_ subset (4, 25). Our data are consistent with these reports, and once again showed that ECP induced an increase of T_regs_ and the CD39^+^ T_reg_ subsets within the first three ECP cycles (= six ECP treatments) of our ECP treatment schedule. We also showed that additional ECP treatment, up to ten cycles, reduced the percentage of CD62L^+^ T_regs_ compared to those observed in our previous results in which three ECP cycles (= six ECP treatments) did not show a reduction of CD62L^+^ T_regs_ (4). The CD62L expression of T_regs_ has been associated with optimal suppressive function of these cells (26, 27). Although CD62L^+^ and CD62L^-^ T_regs_ have been shown to be equally anergic and suppressive upon *in vitro* stimulation, only the CD62L^+^ T_regs_ protect against lethal acute graft-versus-host disease after bone marrow transplantation (27, 28). The reduction in the fraction of CD62L^+^ T_regs_ after the fifth ECP cycle and the reduced CD62L surface expression indicates that T_regs_ shifted from central memory to an activation state (26, 28). Several animal studies have documented that the loss of CD62L expression leads to a reduction in the protective properties of T_regs_ (26, 27, 29). Therefore, we concluded that the number of ECP cycles could be reduced to six treatments, because the loss of CD62L only appears after six treatments.

To evaluate the patient-specific benefit of ECP treatment, we defined classification criteria to calculate the individual immunological improvement. In our study cohort, 72% of the patients responded to five ECP cycles (= ten ECP treatments) according to our immunological profiling of the stimulation of tolerance-inducing cell subsets. However, ECP-induced effects were not detectable with our immunological profile in 28% of our patients. We hypothesized that the immunological changes would not be substantial enough to induce a clinical benefit in these patients. Furthermore, 56% of the ECP-treated patients showed an immunological improvement after no more than three ECP cycles. For these patients a less intense ECP regimen seems to be adequate.

There are several limitations to the current study. First, this is a descriptive study design that bears to risk for biases and a lack of variability of statistical results. Second, the ECP product was not investigated. About 30% of the centers who treat HTx patients with ECP perform quality controls such as measurement of hematocrit, lymphocyte count, monocyte count etc. (information received from Therakos Inc.). Further analyses are not recommended or performed in centers that treat HTx patients with ECP, but identification of laboratory parameters to qualify the ECP product is recommended (30). Third, we investigated ECP effects using the closed, inline THERAKOS CELLEX photopheresis system. Several closed and open offline systems exist, that may have different technical capacities (31). Therefore, ECP products may differ with regard to cellular composition, total cell numbers, apoptotic cell content, the presence of psoralen photoadducts and excipients (32, 33).

In summary, our study described the changes of tolerance-inducing cell subsets during and after ECP treatment of HTx-patients. Compared to that in previous studies, the DC subsets were analyzed in detail, which revealed an important role in tolerance induction following HTx. The established monitoring tool can distinguish between patients who developed an immunological effect to ECP and patients that did not. Furthermore, we developed classification criteria that may allow identifying patients that would benefit from a reduction or an extension of the number of ECP cycles. Monitoring results including analysis could be available within 4-5 hours following blood withdrawal.

This tool could be helpful for clinicians to monitor ECP treatment for shortening or prolonging the ECP schedule for patients depending on their immunological profile. It is recommended to create a center-specific non-ECP-treated control group dataset consisting of long-term, rejection-free HTx patients and pre-HTx patients to work with a center-specific database for hierarchical clustering. Further, a multicenter study for ECP treatment in HTx patients will be helpful to prove our monitoring tool in the clinical routine.

## Data Availability Statement

The raw data of the flow cytometric measurements and immunological profile grouping supporting the conclusions of this article will be made available by the authors. Patient-specific data can not be transferred to the journal due to reasons of protection of personal data.

## Ethics Statement

The studies involving human participants were reviewed and approved by Ärztekammer Hamburg KdÖR, Hamburg, Germany. The patients/participants provided their written informed consent to participate in this study.

## Author Contributions

Conceptualization: M-TD, MJB, and HR. Methodology: M-TD, KK, FA, MJB, and SK. Validation: MJB, AB, and FA. Formal analysis: M-TD, KK, SK, and SL. Investigation: M-TD and KK. Resources: MJB, FA, and MAB. Data curation: MJB, AB, KK, and SK. Writing—original draft preparation: M-TD and KK. Writing—review and editing: MJB, AB, FA, HR, MAB, SK, and SL. Visualization: M-TD and KK. Supervision: M-TD and MJB. Project administration: M-TD and MJB. All authors contributed to the article and approved the submitted version.

## Conflict of Interest

FA declared the following conflict of interest: Therakos: Honoraria, Research funding. MJB declared the following conflict of interest: Therakos: Honoraria.

The remaining authors declare that the research was conducted in the absence of any commercial or financial relationships that could be construed as a potential conflict of interest.

## Publisher’s Note

All claims expressed in this article are solely those of the authors and do not necessarily represent those of their affiliated organizations, or those of the publisher, the editors and the reviewers. Any product that may be evaluated in this article, or claim that may be made by its manufacturer, is not guaranteed or endorsed by the publisher.
