# Supplementary material for: Immune Monitoring Assay for Extracorporeal Photopheresis Treatment Optimization After Heart Transplantation
Source: Front Immunol. 2021 Aug 10;12:676175. doi: 10.3389/fimmu.2021.676175 (PMC8383491; doi:10.3389/fimmu.2021.676175)
Supplement: Supplementary file 1 [file Image_1.pdf]

Supplementary Figure 1: Exemplary heatmap for a patient with early immunological improvement during ECP treatment in a dataset of patients prior to (pre-HTx) or with long-term heart transplantation (LT-HTx).

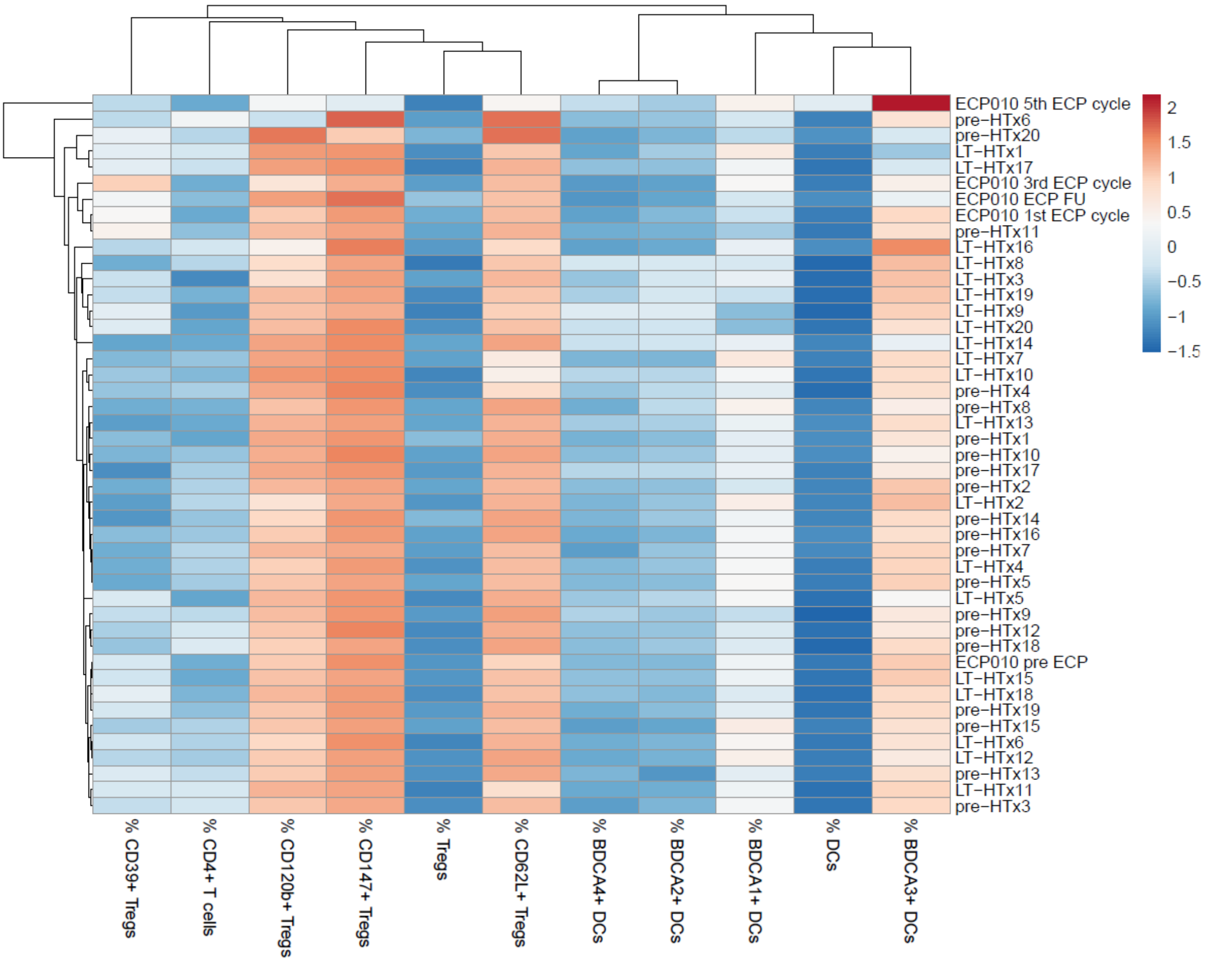

Footnote Supplementary Figure 1: BDCA1/2/3/4, blood dendritic cell antigen 1/2/3/4; CD, cluster of differentiation; DCs, dendritic cells; ECP, extracorporeal photopheresis; FU, follow-up; T_{regs}, regulatory T cells
