# Supplementary material for: Immune Monitoring Assay for Extracorporeal Photopheresis Treatment Optimization After Heart Transplantation
Source: Front Immunol. 2021 Aug 10;12:676175. doi: 10.3389/fimmu.2021.676175 (PMC8383491; doi:10.3389/fimmu.2021.676175)
Supplement: Supplementary file 4 [file Table_1.docx]

**SUPPLEMENTARY TABLE 1:** Immune monitoring of dendritic cell subsets during ECP treatment in HTx patients with ACR/AMR or with a prophylactic ECP treatment.

| Immune parameter | Rejection group  n = 6 | Prophylaxis group  n = 11 |
| --- | --- | --- |
| % DCs/PBMCs  pre-ECP  1st ECP  3rd ECP  5th ECP  ECP FU | 0.8 ± 0.6  0.4 ± 0.2  1.0 ± 0.4  0.8 ± 0.2  1.0 ± 0.3 | 0.4 ± 0.2  0.6 ± 0.2  0.7 ± 0.3  0.7 ± 0.1  0.8 ± 0.1 |
| % BDCA1^+^ DCs/DCs  pre-ECP  1st ECP  3rd ECP  5th ECP  ECP FU | 24.0 ± 10.3  27.3 ± 14.1  28.6 ± 6.8  32.8 ± 2.1  27.7 ± 4.2 | 18.7 ± 6.3  16.6 ± 6.5  16.9 ± 4.8  32.6 ± 3.6  21.8 ± 5.9 |
| % BDCA2^+^ DCs/DCs  pre-ECP  1st ECP  3rd ECP  5th ECP  ECP FU | 42.4 ± 5.0  34.7 ± 13.6  46.2 ± 9.6  54.4 ± 3.3  45.2 ± 4.4 | 43.3 ± 14.8  39.6 ± 15.1  45.2 ± 8.2  52.4 ± 3.6  42.9 ± 10.4 |
| % BDCA3^+^ DCs/DCs  pre-ECP  1st ECP  3rd ECP  5th ECP  ECP FU | 22.6 ± 7.1  27.6 ± 13.1  26.9 ± 7.4  31.8 ± 2.8  23.8 ± 4.8 | 21.5 ± 9.9  14.7 ± 6.9  16.0 ± 5.7  31.8 ± 4.4  20.0 ± 6.2 |
| % BDCA4^+^ DCs/DCs  pre-ECP  1st ECP  3rd ECP  5th ECP  ECP FU | 77.7 ± 5.3  84.7 ± 9.5  88.2 ± 7.5  92.5 ± 1.5  84.0 ± 7.3 | 75.5 ± 7.4  72.7 ± 10.3  78.9 ± 7.9  89.1 ± 5.7  71.0 ± 12.7 |
| MFI BDCA1 [U]  pre-ECP  1st ECP  3rd ECP  5th ECP  ECP FU | 1854 ± 420  2951 ± 2501  2255 ± 313  2245 ± 234  2224 ± 213 | 3499 ± 4508  3118 ± 2338  2413 ± 526  2110 ± 202  2228 ± 233 |
| MFI BDCA2 [U]  pre-ECP  1st ECP  3rd ECP  5th ECP  ECP FU | 1509 ± 229  2145 ± 308  1681 ± 271  1484 ± 59  2216 ± 446 | 1600 ± 341  1424 ± 343  1610 ± 221  1545 ± 76  1925 ± 287 |
| MFI BDCA3 [U]  pre-ECP  1st ECP  3rd ECP  5th ECP  ECP FU | 18309 ± 1797  15771 ± 3345  20877 ± 2618  19259 ± 1002  24337 ± 4374 | 17621 ± 5310  18081 ± 6111  22735 ± 4637  18379 ± 1304  24144 ± 5335 |
| MFI BDCA4 [U]  pre-ECP  1st ECP  3rd ECP  5th ECP  ECP FU | 23467 ± 7952  55173 ± 4630  20502 ± 6269  18054 ± 7674  20673 ± 4750 | 24985 ± 6504  18293 ± 6483  21894 ± 6970  22535 ± 4557  21226 ± 5173 |
| ACR, acute cellular rejection; AMR, antibody-mediated rejection; BDCA1/2/3/4, blood dendritic cell antigen 1/2/3/4; DCs, dendritic cells; ECP, extracorporeal photopheresis; FU, follow up; MFI, mean fluorescence intensity; PBMCs, peripheral blood mononuclear cells; U, unit. | | |
