# Supplementary material for: Immune Monitoring Assay for Extracorporeal Photopheresis Treatment Optimization After Heart Transplantation
Source: Front Immunol. 2021 Aug 10;12:676175. doi: 10.3389/fimmu.2021.676175 (PMC8383491; doi:10.3389/fimmu.2021.676175)
Supplement: Supplementary file 5 [file Table_2.docx]

**SUPPLEMENTARY TABLE 2:** Immune monitoring of T cell and T_reg_ cell subsets during ECP treatment in HTx patients with rejection or with a prophylactic ECP treatment.

| Immune Parameter | Rejection group  n = 6 | Prophylaxis group  n = 11 |
| --- | --- | --- |
| % CD4^+^ T cells/total T cells  pre-ECP  1st cycle ECP  3rd cycle ECP  5th cycle ECP  ECP FU | 27.9 ± 5.6  16.3 ± 7.1  26.0 ± 7.9  24.1 ± 4.3  21.0 ± 9.9 | 19.9 ± 6.0  16.9 ± 4.8  20.9 ± 6.0  23.6 ± 2.6  13.5 ± 3.8 |
| % T_regs_/CD4^+^ T cells  pre-ECP  1st cycle ECP  3rd cycle ECP  5th cycle ECP  ECP FU | 7.8 ± 2.1  9.7 ± 2.6  12.4 ± 3.2  12.8 ± 2.2  14.6 ± 4.1 | 11.0 ± 1.8  12.9 ± 2.9  15.3 ± 2.3  12.2 ± 0.7  19.4 ± 2.9 |
| % CD39^+^ T_regs_/ total T_regs_  pre-ECP  1st cycle ECP  3rd cycle ECP  5th cycle ECP  ECP FU | 38.5 ± 8.4  46.0 ± 22.3  48.8 ± 21.3  32.8 ± 2.9  60.0 ± 25.6 | 38.6 ± 20.0  46.9 ± 23.2  57.7 ± 20.1  32.0 ± 8.4  52.2 ± 19.6 |
| % CD62L^+^ T_regs_/ total T_regs_  pre-ECP  1st cycle ECP  3rd cycle ECP  5th cycle ECP  ECP FU | 79.7 ± 5.2  71.7 ± 11.2  76.7 ± 9.8  65.0 ± 14.2  83.1 ± 6.7 | 75.8 ± 14.4  71.7 ± 15.4  74.6 ± 18.9  51.2 ± 10.6  79.2 ± 13.8 |
| % CD120b^+^ T_regs_/ total T_regs_  pre-ECP  1st cycle ECP  3rd cycle ECP  5th cycle ECP  ECP FU | 73.3 ± 12.5  67.6 ± 15.0  69.1 ± 10.4  68.7 ± 11.5  66.4 ± 7.5 | 68.7 ± 14.2  66.4 ± 16.4  72.6 ± 12.0  69.2 ± 14.2  64.7 ± 11.3 |
| % CD147^+^ T_regs_/ total T_regs_  pre-ECP  1st cycle ECP  3rd cycle ECP  5th cycle ECP  ECP FU | 93.0 ± 10.4  88.1 ± 17.2  88.1 ± 9.5  94.4 ± 2.6  89.1 ± 5.7 | 94.8 ± 5.7  92.3 ± 8.1  92.4 ± 3.8  94.3 ± 2.8  90.6 ± 7.2 |
| MFI CD39 [U]  pre-ECP  1st cycle ECP  3rd cycle ECP  5th cycle ECP  ECP FU | 770 ± 145  931 ± 248  849 ± 223  1112 ± 257  758 ± 195 | 864 ± 239  814 ± 267  923 ± 190  657 ± 160  753 ± 410 |
| MFI CD62L [U]  pre-ECP  1st cycle ECP  3rd cycle ECP  5th cycle ECP  ECP FU | 9113 ± 2143  6146 ± 2154  5616 ± 1320  5131 ± 1389  7938 ± 2307 | 8329 ± 2691  6216 ± 2735  6177 ± 1416  5952 ± 1526  7821 ± 832 |
| MFI CD120b [U]  pre-ECP  1st cycle ECP  3rd cycle ECP  5th cycle ECP  ECP FU | 1151 ± 204  1237 ± 130  1278 ± 281  1516 ± 30  1148 ± 208 | 1226 ± 351  1188 ± 205  1272 ± 205  1486 ± 25  1070 ± 77 |
| MFI CD147 [U]  pre-ECP  1st cycle ECP  3rd cycle ECP  5th cycle ECP  ECP FU | 1475 ± 282  1752 ± 336  1462 ± 221  1369 ± 5  1288 ± 139 | 1416 ± 357  1509 ± 496  1545 ± 314  1389 ± 61  1302 ± 149 |
| CD, cluster of differentiation; ECP, extracorporeal photopheresis; FU, follow up; MFI, mean fluorescence intensity; T_regs_, regulatory T cells; U, unit | | |
|  |  |  |
